# Plasmodium Infection Induces Dyslipidemia and a Hepatic Lipogenic State in the Host through the Inhibition of the AMPK-ACC Pathway

**DOI:** 10.1038/s41598-019-51193-x

**Published:** 2019-10-11

**Authors:** George Eduardo Gabriel Kluck, Camila Hübner Costabile Wendt, Guinever Eustaquio do Imperio, Maria Fernanda Carvalho Araujo, Tainá Correa Atella, Isabella da Rocha, Kildare Rocha Miranda, Georgia Correa Atella

**Affiliations:** 1 Laboratory of Lipid and Lipoproteins Biochemistry, Leopoldo de Meis Institute of Medical Biochemistry, Federal University of Rio de Janeiro, Rio de Janeiro, Brazil; 2 Laboratory of Cellular Ultrastructure Hertha Meyer, Carlos Chagas Filho Institute of Biophysics, Federal University of Rio de Janeiro, Rio de Janeiro, Brazil; 3 Laboratory of Translational Endocrinology, Carlos Chagas Filho Institute of Biophysics, Federal University of Rio de Janeiro, Rio de Janeiro, Brazil; 4 Laboratory of Comparative Neurobiology and Development, Carlos Chagas Filho Institute of Biophysics, Federal University of Rio de Janeiro, Rio de Janeiro, Brazil

**Keywords:** Fatty acids, Infection

## Abstract

Malaria is a major parasitic disease of humans and is a health public problem that affects more than 100 countries. In 2017, it caused nearly half a million deaths out of 219 million infections. Malaria is caused by the protozoan parasites of the genus *Plasmodium* and is transmitted by female mosquitoes of the genus *Anopheles*. Once in the bloodstream, *Plasmodium* merozoites invade erythrocytes and proliferate until the cells lyses and release new parasites that invade other erythrocytes. Remarkably, they can manipulate the vertebrate host’s lipid metabolism pathways, since they cannot synthesize lipid classes that are essential for their development and replication. In this study, we show that mice infected with *Plasmodium chabaudi* present a completely different plasma profile from control mice, with marked hyperproteinemia, hypertriglyceridemia, hypoglycemia, and hypocholesterolemia. In addition, white adipose and hepatic tissue and analyses from infected animals revealed the accumulation of triacylglycerol in both tissues and free fatty acids and free cholesterol in the liver. Hepatic mRNA and protein expression of key enzymes and transcription factors involved in lipid metabolism were also altered by *P. chabaudi* infection, leading to a lipogenic state. The enzyme 5′ AMP-activated protein kinase (AMPK), a master regulator of cell energetic metabolism, was also modulated by the parasite, which reduced AMPK phosphorylation levels upon infection. Pretreatment with metformin for 21 days followed by infection with *P. chabaudi* was effective in preventing infection of mice and also lowered the hepatic accumulation of lipids while activating AMPK. Together, these results provide new and important information on the specific molecular mechanisms induced by the malaria parasite to regulate hepatic lipid metabolism in order to facilitate its development, proliferation, and lifespan in its vertebrate host.

## Introduction

Malaria is a major parasitic disease of humans^1^. It is a life-threatening disease^2^ and yet is considered to be a neglected disease and public health problem affecting more than 100 countries worldwide^3^. In 2017, it caused nearly half a million deaths out of 219 million infections. More than three billion people are at risk of being infected and developing malarial disease at this very moment^4^.

Malaria is an infectious disease caused by protozoan parasites of the genus *Plasmodium* and is transmitted by female mosquitoes of the genus *Anopheles*^5^. As a multifactorial disease, its clinical progression depends on several host factors, including age, previous exposure to infection, nutritional status, genetics of the host and parasite, geographic location, and socioeconomic status^6–8^. There are some events, however, that are fundamental to malarial pathogenesis: (1) the massive release of proinflammatory cytokines induced by the rupture of infected erythrocytes, (2) the adhesion of infected red blood cells (RBCs) in capillaries, and (3) the rupture and removal of infected RBCs by splenic macrophages. Together, these phenomena are responsible for the major syndromes associated with malaria, such as systemic inflammation, anemia, metabolic acidosis, and placental and cerebral malaria^6–8^. In the liver, *Plasmodium* infection is associated with portal tract inflammation^9^, cholestasis^9^, RBCs sequestration, and hepatocytes necrosis^10^. Therefore, patients with malaria usually present with hepatomegaly, jaundice, an increased hepatic load of RBCs, and elevated serum levels of hepatic enzymes^11^.

The liver is a central metabolic organ and is the main regulator of glucose and lipid metabolism, masterfully performing gluconeogenesis, β-oxidation, lipogenesis, and uptake and secretion of lipoproteins^12^. The enzyme 5′ AMP-activated protein kinase (AMPK) is a key regulator of liver lipid metabolism^13^. When activated, AMPK phosphorylates and inhibits the enzymes acetyl-CoA carboxylase (ACC) and 3-hydroxy-3-methylglutaryl-coenzyme A reductase (HMGCR), decreases fatty acid synthase (FAS) expression, and activates malonyl-CoA carboxylase, which leads to a reduction in fatty acid and cholesterol synthesis^14–16^. The Inhibition of ACC also stimulates fatty acids’ oxidation through a decrease of malonyl-CoA^17,18^, leading to an increased activity of carnitine palmitoyl transferase 1 (CPT1)^19^.

Considering that the metabolic consequences and the specific hepatic molecular mechanisms underlying the pathogenesis of malaria are still unclear, we aimed to investigate changes in lipid metabolism during *Plasmodium chabaudi* infection in mice in order to better understand their importance to the host–parasite interaction and to the outcome of the infection.

## Results

### *P. chabaudi* infection induces plasma dyslipidemia and altered profile of cytokines in swiss mice

**Figure 1:**
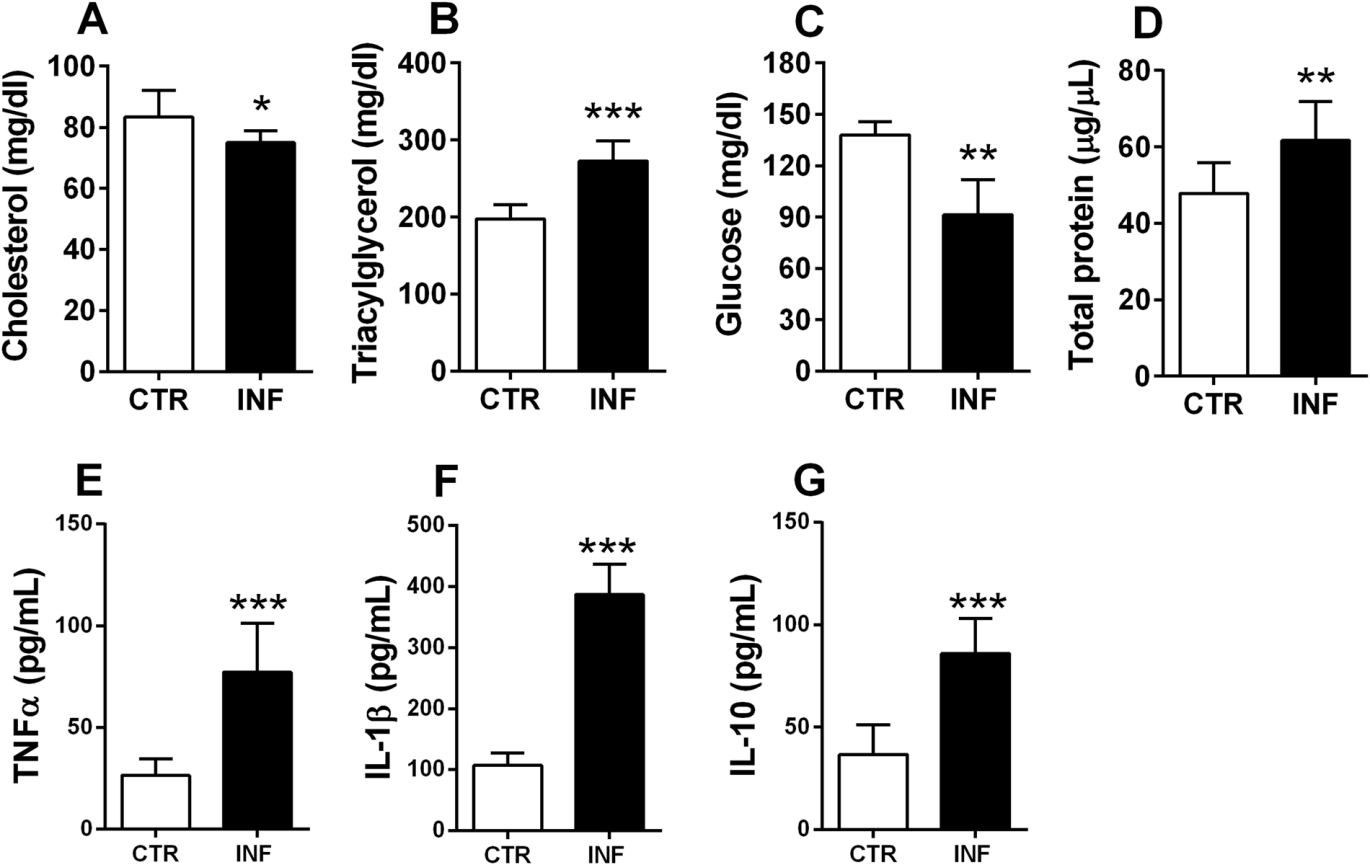
Plasma analysis of *P. chabaudi* infected mice. (**A**) Total cholesterol, (**B**) triacylglycerol, and (**C**) glucose concentrations obtained using enzymatic colorimetric assays. (**D**) Total protein concentration measured by the Lowry method. (**F**–**H**) TNFα, IL-1β, and IL-10 concentration measured by ELISA. White bars indicate control (CTR, *n* = 14) and black bars indicate infected (INF, *n* = 14) groups. Statistical analyses: unpaired Student’s *t*-test. Values are presented as mean ± standard error. ^*^p < 0.05, ^**^p < 0.01, and ^***^p < 0.001.

To evaluate the plasma profiles of infected mice, plasma samples were analyzed for total cholesterol, triacylglycerol (TAG), glucose, total protein, tumor necrosis factor alpha (TNFα), interleukin (IL)-1β, and IL-10 concentrations (Fig. 1). Total cholesterol and glucose levels decreased significantly in infected mice compared to controls (*p* < 0.05 and *p* < 0.01, respectively; Fig. 1A,C), whereas TAG and total protein levels increased in infected animals (*p* < 0.001 and *p* < 0.01, respectively; Fig. 1B,D). TNFα, IL-1β, and IL-10 concentrations increased significantly in infected mice compared to control group (*p* < 0.0001, *p* < 0.0001 and *p* < 0.0001, respectively; Fig. 1E–G). Together, these results revealed marked changes in plasma lipids, protein, and cytokines in infected versus control mice, suggesting dyslipidemia and inflammation response induced by infection.

### *P. chabaudi* promotes changes in lipid classes in the liver and white adipose tissue (WAT) of infected mice

**Figure 2:**
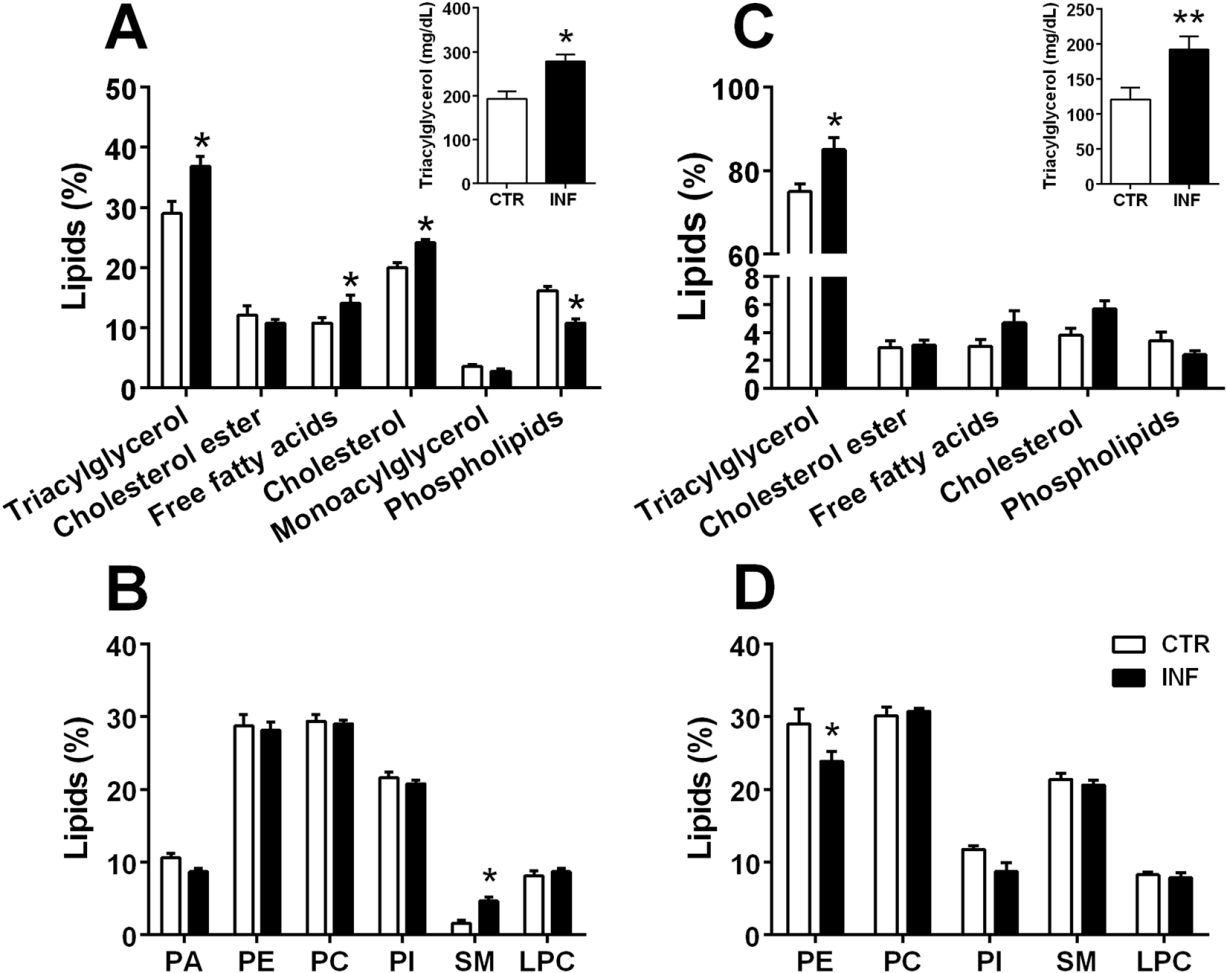
Lipid profile analysis in the liver (**A**,**B**) and white adipose tissue (WAT; **C**,**D**) of mice infected with *P. chabaudi*. The graphs show lipid classes obtained through thin-layer chromatography and percentages determined by densitometric analysis. Hepatic (**A**) and WAT (**C**) analyses of neutral lipids showing the percentage of triacylglycerol (TAG), cholesterol ester (CHOE), free fatty acid (FFA), cholesterol (CHO), monoacylglycerol (MAG), and phospholipid (PHO) in respective tissues of control (CTR, *n* = 14) versus infected (INF, *n* = 14) mice. Insets in (**A**,**C**) upper right represent TAG concentration obtained using enzymatic colorimetric assays. Hepatic (**B**) and WAT (**D**) analyses of phospholipids showing the percentage of phosphatidic acid (PA), phosphatidylethanolamine (PE), phosphatidylcholine (PC), phosphatidylinositol (PI), sphingomyelin (SM), and lysophosphatidylcholine (LPC) in respective tissues of CTR versus INF animals. Statistical analyses: multiple *t*-test. Values are presented as mean ± standard error. ^*^p < 0.05 and ^**^p < 0.01.

Analysis of hepatic and WAT lipid profiles was performed to investigate the possible alterations in lipid classes during *Plasmodium* infection. As shown in Fig. 2A,B, hepatic TAG (*p* < 0.05), free cholesterol (*p* < 0.05), free fatty acids (*p* < 0.05), and sphingomyelin (SM; *p* < 0.05) levels increased in infected animals relative to controls, whereas the WAT of infected animals exhibited elevated TAG (*p* < 0.05; Fig. 2C) and decreased phosphatidylethanolamine (PE; *p* < 0.05; Fig. 2D), with no significant differences found in the other lipid classes. Enzymatic colorimetric assays were used to confirm the hepatic and WAT changes in the TAG concentration and validate the increase of this lipid class in both tissues in the infected versus control groups (inset in Fig. 2A,C). Together, these data indicate that *P. chabaudi* infection induces an increase in lipid accumulation in tissues that are critical to lipid metabolism in mice.

### *P. chabaudi* infection leads to an altered hepatic expression of genes associated with lipid metabolism

**Figure 3:**
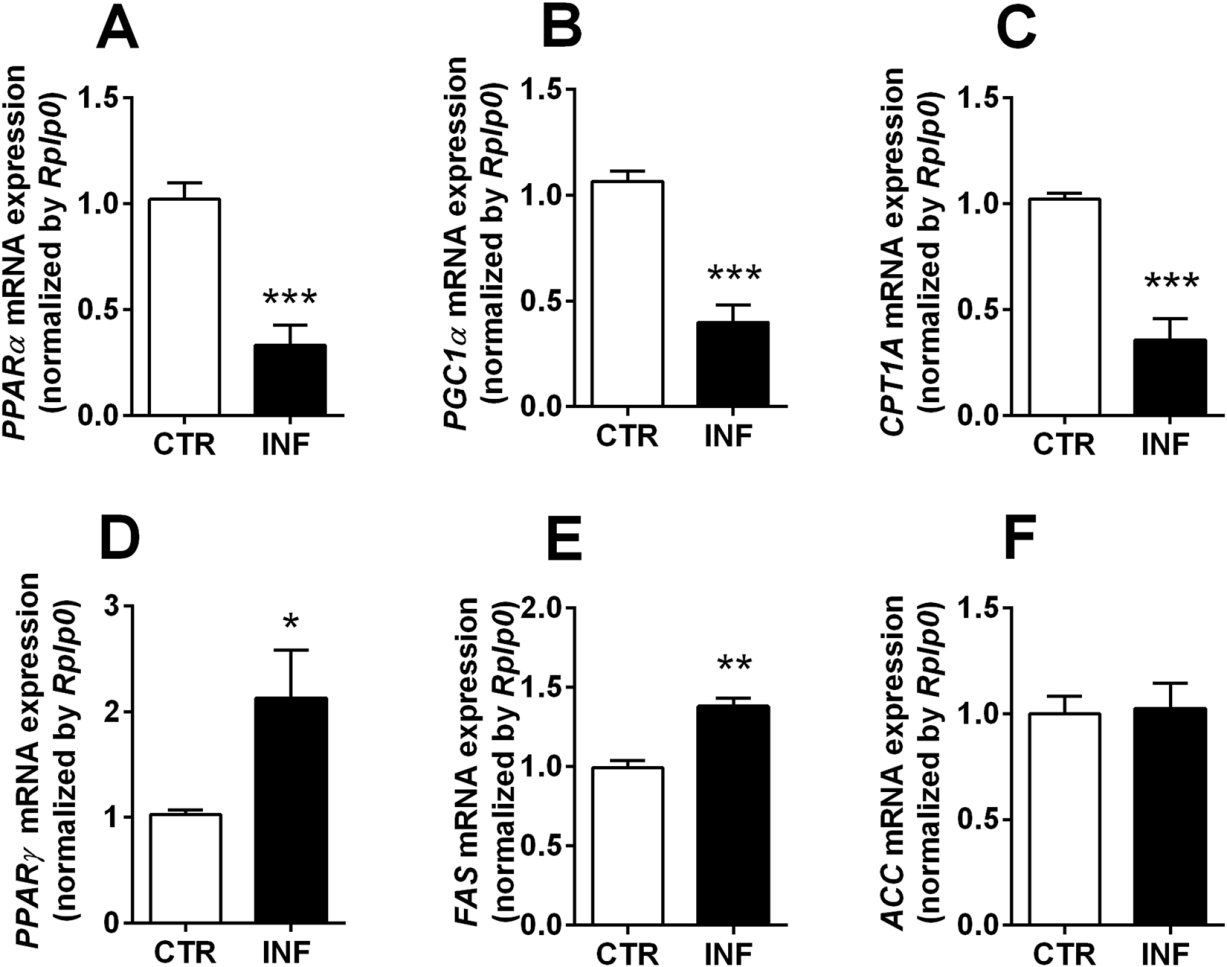
Gene expression of key enzymes and transcription factors involved in lipid metabolism in the liver of mice infected with *P. chabaudi*. *PPAR-α* (**A**), *PGC-1α* (**B**), *CPT1-A* (**C**), *PPAR-γ* (**D**), *FAS* (**E**), and *ACC* (**F**) gene expression analysis. *Rplp0* was used as an endogenous control. White bars indicate control (CTR, *n* = 14) and black bars indicate infected (INF, *n* = 14) groups. Statistical analyses: unpaired Student’s *t*-test. Values are presented as mean ± standard error. ^*^p < 0.05, ^**^p < 0.01, and ^***^p < 0.001.

After demonstrating that *Plasmodium* infection in mice induces a wide spectrum of lipid changes, including alterations in plasma lipids and hepatic and WAT lipid classes, we next hypothesized that *P. chabaudi* specifically impairs the gene expression of key enzymes and transcription factors involved in lipid degradation or biosynthesis (Table 1). In this respect, the transcription factors peroxisome proliferator-activated receptor-α (PPAR-α) and PPAR gamma coactivator-1α (PGC-1α) and the β-oxidation enzyme CPT1-A were chosen for investigation as genes involved in lipid degradation pathways; all decreased significantly in infected mice (*p* < 0.001; Fig. 3A–C). The transcription factor PPAR-γ and the enzymes FAS and ACC were chosen to assess the mediators of lipid biosynthesis pathways; PPAR-γ and FAS levels increased in infected mice (*p* < 0.05 and *p* < 0.01; Fig. 3D,E), with no significant difference in the ACC gene expression (Fig. 3F). Together, these findings demonstrate that malaria induces significant changes in hepatic gene expression, whose combination points to an increase in lipogenic and a decrease in lipolytic pathways.

**Table 1:** Primers sequence used for gene expression assessment.

Primer	Forward	Reverse
*Acaca*	5′-AACATCCCCACGCTAAACAG-3′	5′-CTGACAAGGTGGCGTGAAG-3′
*Cpt1a*	5′-AGTGGCCTCACAGACTCCAG-3′	5′-CCCATGTTGTACAGCTTCC-3′
*Fasn*	5′-CCCTTGATGAAGAGGGATCA-3′	5′-GAACAAGGCGTTAGGGTTGA-3′
*Ppargc1a*	5′-ATGTGTCGCCTTCTTGCTCT-3′	5′-ATCTACTGCCTGGGGACCTT-3′
*Ppara*	5′-CAGTGGGGAGAGAGGACAGA-3′	5′-AGTTCGGGAACAAGACGTTG-3′
*Pparg*	5′-GATGGAAGACCACTCGCATT-3′	5′-AACCATTGGGTCAGCTCTTG-3′

*Acaca*: acetyl-coa carbolixase; *Cpt1a*: carnitine palmitoyl transferase; *Fasn*: fatty acid synthase; *Ppargc1a*: peroxisome proliferator-activated receptor gamma coactivator 1-alpha; *Ppara*: peroxisome proliferator activated receptor alpha; *Pparg*: peroxisome proliferator activated receptor gamma.

### *P. chabaudi* infection modifies the phosphorylation levels and protein expression of hepatic enzymes and transcription factors involved in lipid metabolism

**Figure 4:**
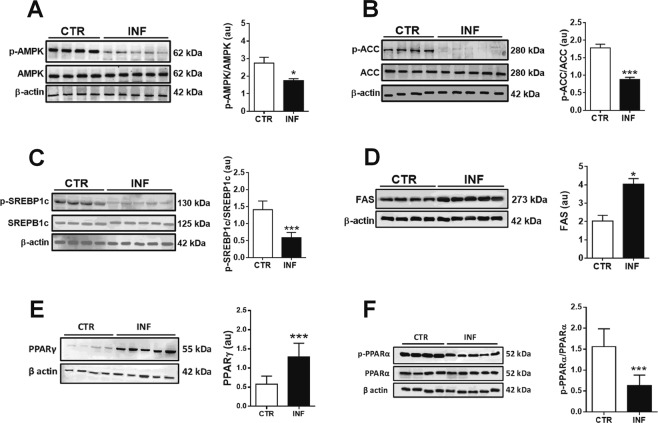
Protein expression of key enzymes and transcription factors involved in lipid metabolism in the liver of mice infected with *P. chabaudi*. Representative immunoblots and the quantification of phosphoprotein (p) and/or total protein levels of AMPK (**A**), ACC (**B**), SREBP-1c (**C**), FAS (**D**), PPARα (**E**), and PPARγ (**F**). β-Actin was used as loading controls. Images represents cropped blots of the same membrane. Cropped slices are showed on Supplementary Information (S1). White bars indicate control (CTR, *n* = 14) and black bars indicate infected (INF, *n* = 14) groups. Statistical analyses: unpaired Student’s *t*-test. Values are presented as mean ± standard error. ^*^p < 0.05 and ^***^p < 0.001.

AMPK is a master regulator of cellular energetic metabolism^20^. When phosphorylated, this kinase is activated and also uses phosphorylation to downregulate anabolic and stimulate catabolic pathways in order to restore cellular energy balance^20^. Thus, we hypothesized that hepatic phosphorylation levels of AMPK and its downstream targets would be altered by *P. chabaudi* infection in these animals. We then demonstrated that AMPK phosphorylation levels decrease significantly upon infection (*p* < 0.05; Fig. 4A and Supplementary S1A), which is accompanied by decreased phosphorylation of ACC, the transcription factor sterol regulatory element binding protein-1c (SREBP-1c), and PPARα (*p* < 0.001; Fig. 4B,C,F, respectively and Supplementary S1B,C,F). Also, there was an increased protein expression of FAS and PPARγ (*p* < 0.05; Fig. 4D,E; Supplementary S1D,E).

### Pretreatment with metformin reduces *P. chabaudi* infection in mice and protects the liver against steatosis

**Figure 5:**
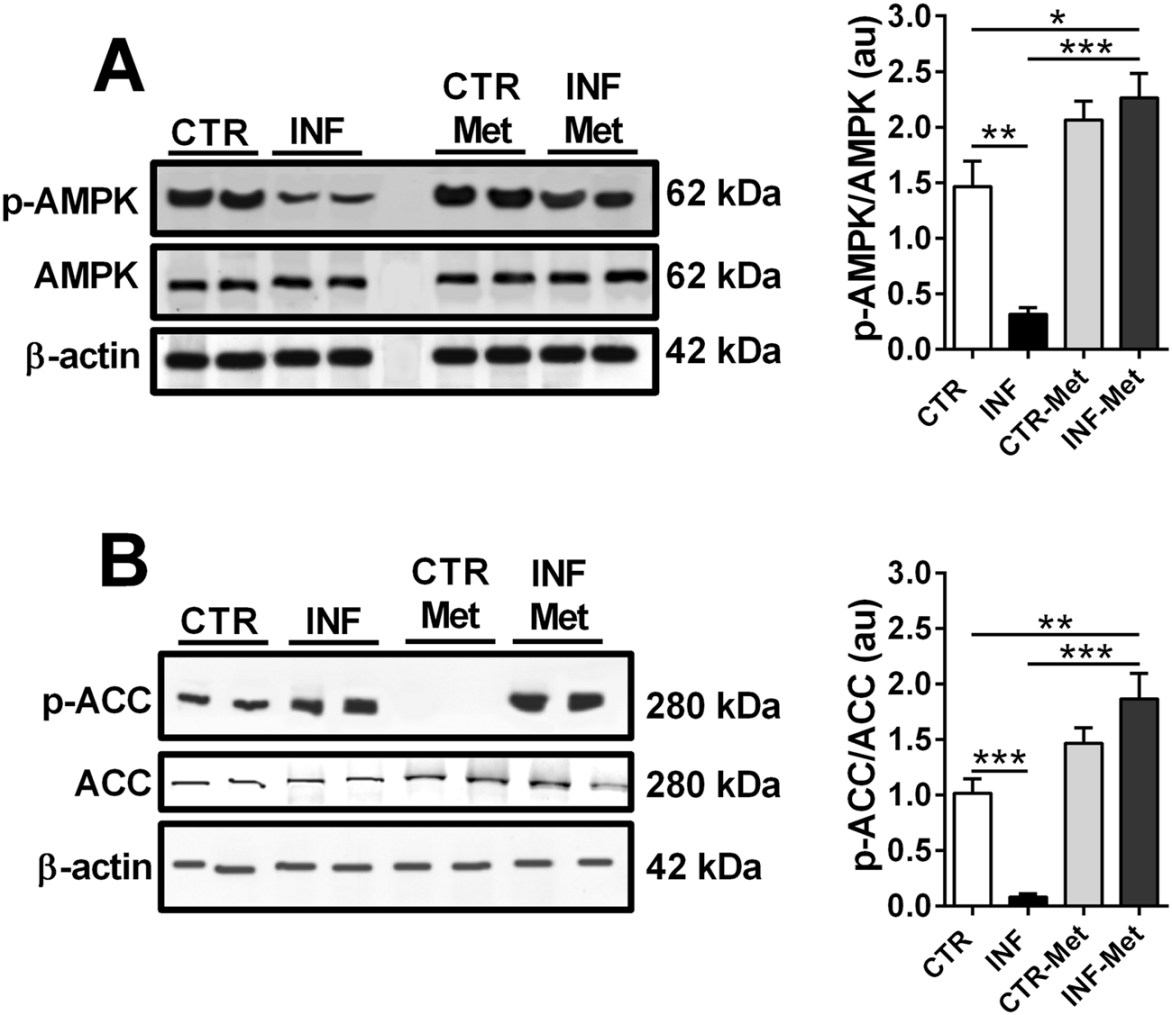
Effect of metformin pretreatment on the protein expression of AMPK and ACC in the liver of mice infected with *P. chabaudi*. Representative immunoblots and quantification of phosphorylation levels of AMPK (**A**) and ACC (**B**). Images represents cropped blots of the same membrane. Cropped slices are showed on Supplementary Information (S2). White bars indicating control (CTR, *n* = 9), black bars indicate infected (INF, *n* = 9), light gray bars indicate metformin pretreated control (CTR-Met, *n* = 9), and dark gray bars indicate metformin pretreated infected (INF-Met, *n* = 7) groups. Statistical analyses: one-way ANOVA followed by Dunnett’s multiple comparisons test. Values are presented as mean ± standard error. ^***^p < 0.001.

**Figure 6:**
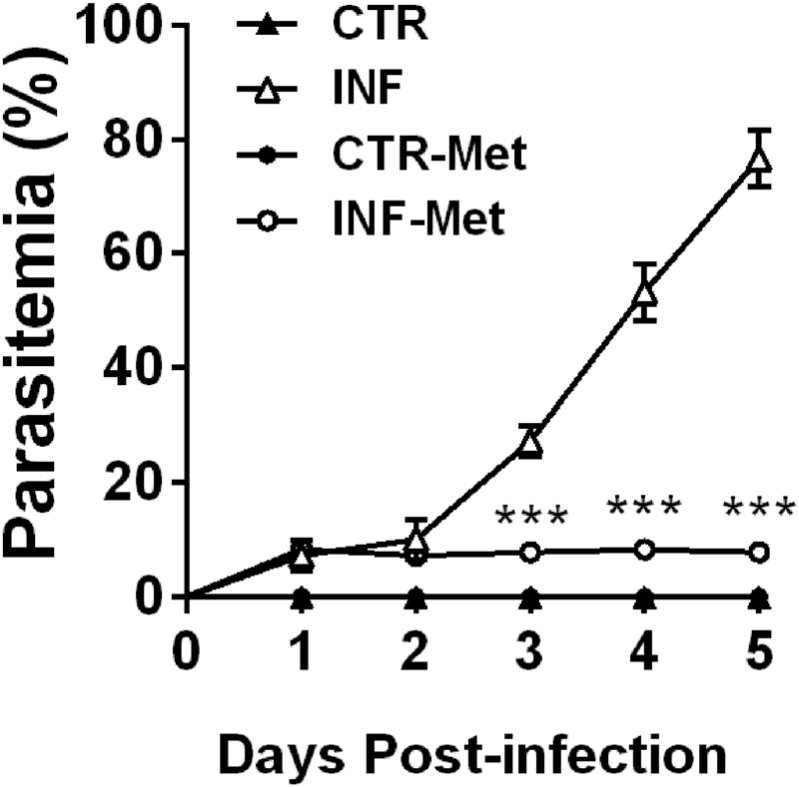
Effect of metformin pretreatment on the effectiveness of *P. chabaudi* infection in mice. Percentage parasitemia assessed through evaluation of blood smears every day until the 5th day after infection. Closed triangles represent controls (*n* = 9), closed circles represent metformin pretreated controls (*n* = 9), open triangles represent the infected group (*n* = 9), and open circles represent the infected group pretreated with metformin (*n* = 7). Statistical analysis: two-way ANOVA followed by Tukey’s multiple comparisons test. Values are presented as mean ± standard error. ^***^p < 0.001.

**Figure 7:**
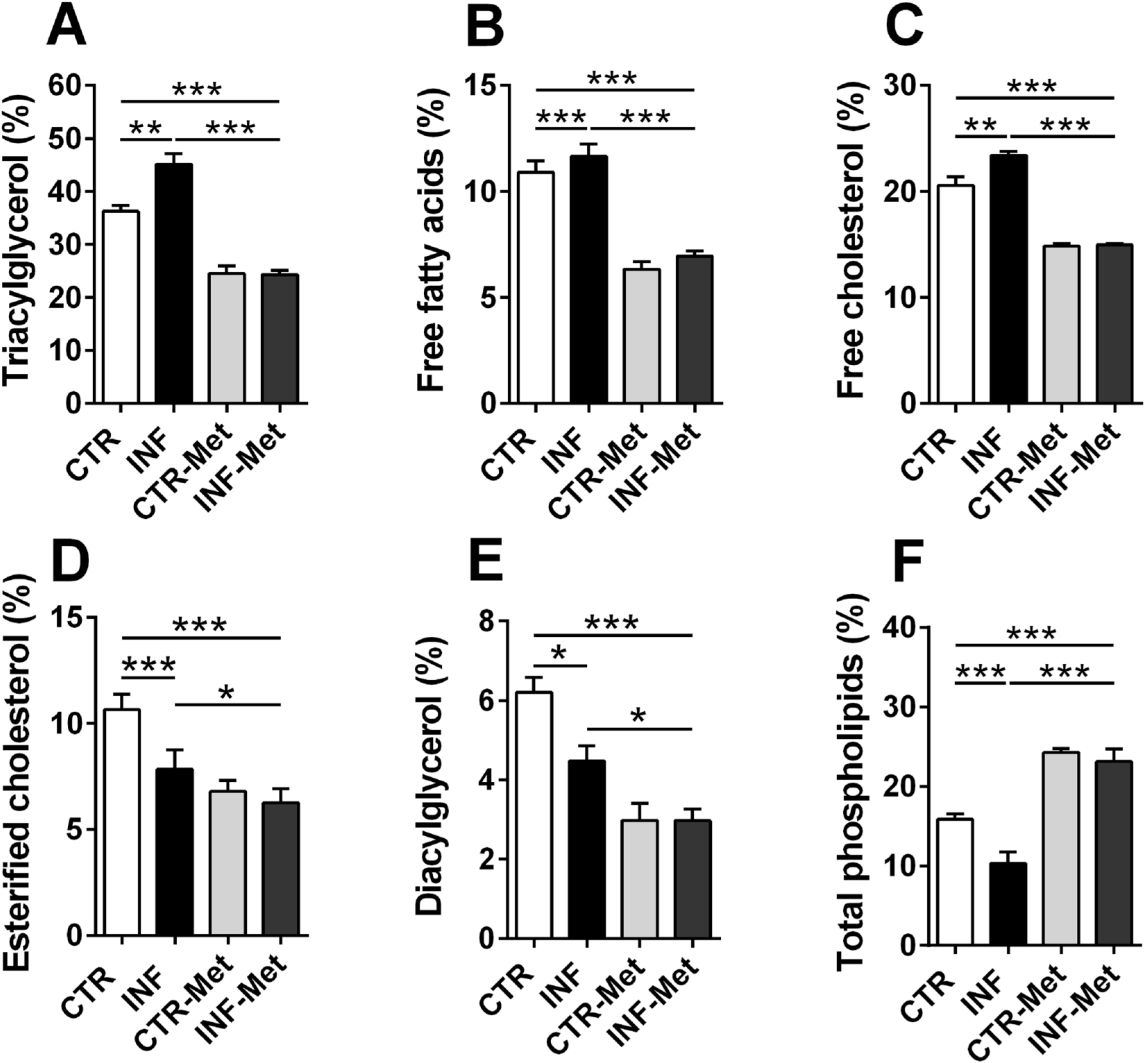
Effect of metformin pretreatment on the hepatic lipid profile of mice infected with *P. chabaudi*. The graphs show lipid classes obtained by thin-layer chromatography and percentages determined using densitometric analysis. White bars indicate controls (CTR, *n* = 9), black bars indicate the infected group (INF, *n* = 9), light gray bars indicate the metformin pretreated controls (CTR-Met, *n* = 9), and dark gray bars indicate the metformin pretreated infected group (INF-Met, *n* = 7). Statistical analyses: one-way ANOVA followed by Dunnett’s multiple comparisons test. Values are presented as mean ± standard error. ^**^p < 0.01 and ^***^p < 0.001.

**Figure 8:**
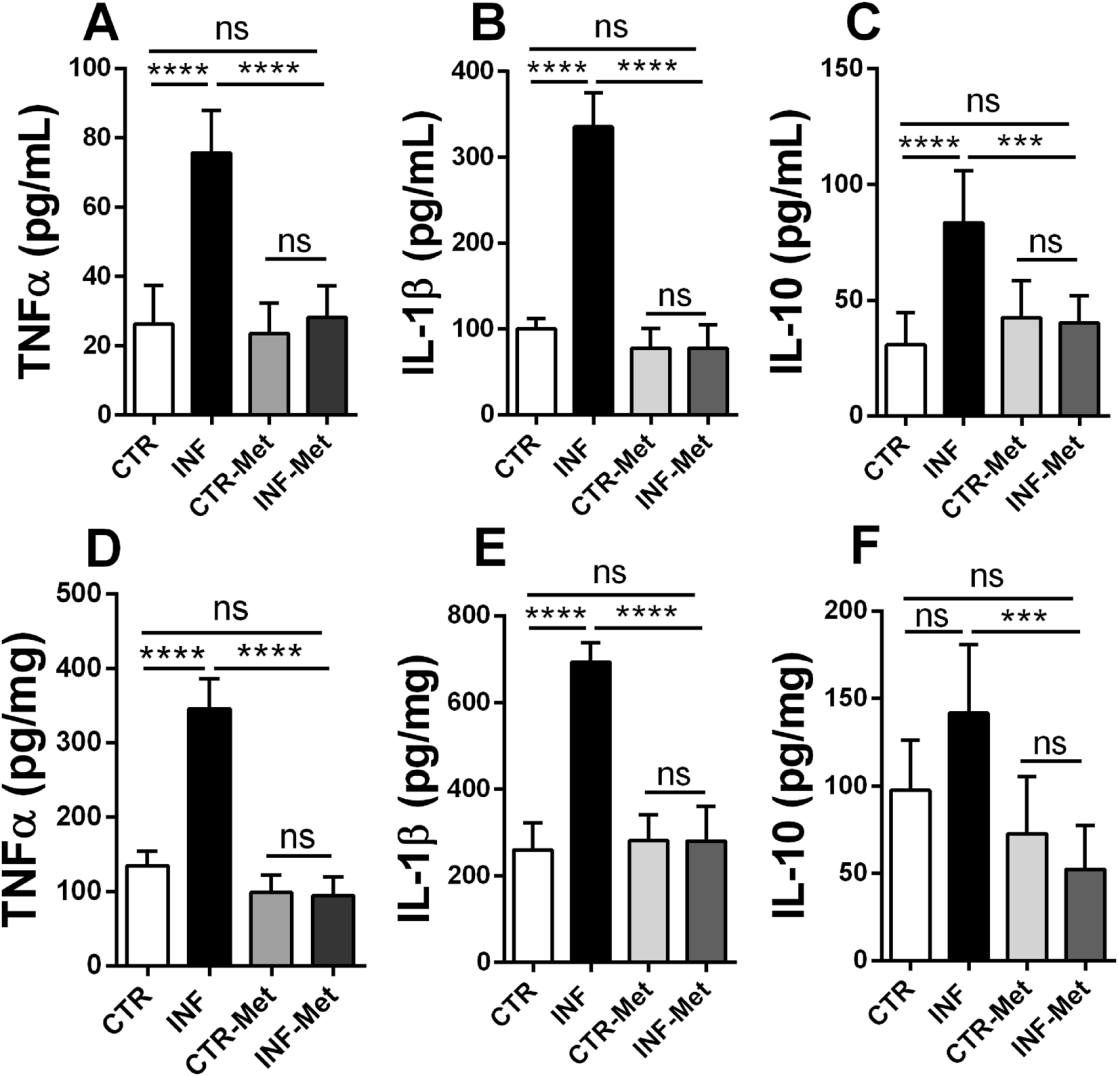
Effect of pretreatment of metformin on cytokines profile measured on plasma and liver of Swiss mice. The graphs shows the concentration of TNFα, IL-1β, and IL-10 obtained by ELISA assay. White bars indicate controls (CTR, *n* = 9), black bars indicate the infected group (INF, *n* = 9), light gray bars indicate the metformin pretreated controls (CTR-Met, *n* = 9), and dark gray bars indicate the metformin pretreated infected group (INF-Met, *n* = 7). Statistical analyses: one-way ANOVA followed by Dunnett’s multiple comparisons test. Values are presented as mean ± standard error. ^**^p < 0.01 and ^***^p < 0.001.

In order to validate the critical role of AMPK-mediated lipid metabolism alteration induced by infection, mice were pretreated with the AMPK activator metformin (1,1-dimethyl biguanide) prior to *P. chabaudi* inoculation (see Materials and Methods). Metformin pretreatment completely reversed the decrease in hepatic AMPK phosphorylation levels observed following *P. chabaudi* infection (*p* < 0.001; Fig. 5A and Supplementary S2A). Similarly, metformin pretreatment abolished the decrease and even increased the hepatic phosphorylation of ACC (a downstream target of AMPK) in the infected metformin group compared to the infected (*p* < 0.001) and control (*p* < 0.001; Fig. 5B and Supplementary S2B) groups. Remarkably, while infected mice presented a gradual and marked increase in parasitemia percentage, reaching 80% on the 5th day after infection (Fig. 6), pretreatment with metformin resulted in a dramatic reduction of the parasitemia percentage from the 3rd to the 5th day after infection (*p* < 0.001; Fig. 6). This significant drop in the infection rate was also accompanied by reduced clinical signs of infection, such as lethargy, piloerection, hypothermia, starvation, and anemia were all abrogated in the pretreated infected animals compared to the untreated infected mice. In addition, pretreatment with metformin induced a decrease in the percentage of all hepatic lipid classes both in infected and in noninfected mice (*p* < 0.001; Fig. 7), except for total phospholipids, which were higher than in the nontreated groups (*p* < 0.001; Fig. 7). Pretreatment with metformin caused a significantly decrease on TNFα, IL-1β and IL-10 both in plasma and liver of infected mice compared to control group (Fig. 8A–F).

## Discussion

In this study, we demonstrated that *P. chabaudi* infection induces severe changes in the vertebrate host’s lipid metabolism that manifest as plasma, hepatic, and WAT metabolic modifications. These changes are mediated by AMPK downregulation and the subsequent activation of pathways that lead to increased lipid biosynthesis and accumulation. Pretreatment with metformin reversed hepatic changes induced by infection, suggesting the establishment of a protective metabolic state that inhibited pathogen proliferation and consequently dissemination in the mammal host.

The balance between host pro- and anti-inflammatory immune responses plays a critical role in determining the outcome of *Plasmodium* infection. A weak pro-inflammatory response may result in unregulated replication of parasites, while an active pro-inflammatory response may cause tissue damage, such as occurs in severe malaria syndromes, including cerebral malaria and multi-organ failure. Here, we showed an increased TNFα, IL-1β, and IL-10 in plasma and liver of infected mice compared to control group (Fig. 1). High levels of plasma TNFα have been associated with anemia and high density *P. falciparum* infection^21^, as well as with other severe malaria complications such as renal failure^22^. Data from mouse models of malaria indicates that IL-10 is required to protect host tissue from inflammation, but may also promote growth of parasites and associated disease manifestations. Thus, IL-10 appears to play a critical role in regulating the effects of TNF during malaria, but at the same time, IL-10 may promote high-density infections that result in other complications, including accumulation of infected red blood cells in tissue that can cause hypoxia and direct damage to the vasculature^23,24^.

*P. chabaudi* promoted hypocholesterolemia, hypertriglyceridemia, hypoglycemia, hyperproteinemia, and altered profile of cytokines, which clearly indicated plasma lipid dysregulation and increased proinflammatory response that is consistent with previous studies^25–29^. However, we also discovered that these plasma changes are accompanied by alterations in the lipid profile of WAT and with increased percentages of TAG and SM; to our knowledge, this is the first time that these effects have been reported in the literature. Considering that infected erythrocytes adhere to capillary endothelial cells in multiple tissues (including WAT^30^), it is possible that the parasite acts indirectly on the adipocytes from the endothelium and stimulates lipid accumulation to benefit from these lipids for its development and multiplication in the host. Further studies are needed to better understand the molecular pathways involved in this regulation and to address its relevance to host–parasite interactions.

Our study was focused on the liver because this tissue possess the complete set of enzymatic machinery necessary to synthesize lipids, since it is the main organ for lipid exchange in the body^31,32^. Liver from infected mice exhibited increased percentages of TAG, free fatty acids, and free cholesterol, suggestive of hepatic dyslipidemia, and our quantitative polymerase chain reaction (qPCR) results corroborated this by revealing a decreased hepatic mRNA expression of PPAR-α, PGC-1α, and CPT1-A during infection. PPAR-α is the most abundant transcription factor in healthy livers. It is responsible for activating fatty acid uptake and oxidation, and PPAR-α hepatocyte-specific knockout impairs free fatty acid homeostasis and affects the entire body in fed or fasting mice^33^. PGC-1α is a transcriptional coactivator induced by fasting, and it activates fatty acid oxidation and gluconeogenesis via PPAR-α, FoxO1, and HNF-4α stimulation^34–36^. CPT1-A is a key enzyme in the β-oxidation process and is regulated not only by malonyl-CoA, but also by thyroid hormone and insulin^37,38^. Together, our results all point to a lower fatty acid oxidation state in infected compared to healthy livers.

In contrast, we observed an increased hepatic mRNA and protein expression of PPAR-γ and FAS in infected compared to control mice. FAS is key enzyme in the process of fatty acid synthesis, and its knockout in mice is lethal, indicating a crucial role for *de novo* lipogenesis in embryonic development^39^. PPAR-γ is a transcriptional regulator of hepatic fatty acid storage, and its liver-specific knockout in obese mice reduces lipid accumulation^40^. Thus, the increase in PPAR-γ and FAS gene expression implies increased lipid biosynthesis during infection.

The enzyme AMPK is a central regulator of the biosynthesis and degradation of lipids, carbohydrates, and proteins^41^. Here we showed that hepatic AMPK is strongly regulated during *Plasmodium* infection, which decreases AMPK phosphorylation and leads to the activation of an anabolic state (Figs 4A and S1A). ACC, SREBP-1c, and PPARα are also essential in controlling lipid synthesis/degradation pathways, and they are targets for AMPK phosphorylation^42,43^. *Plasmodium* infection decreased ACC, and SREBP-1c phosphorylation (i.e., induced their activation) (Figs 4B,C and S1B,C) and decreased PPARα phosphorylation (i.e., decrease their activation) (Figs 4E and S1E). In addition, infection increased the protein expression of FAS and PPARγ (Figs 4D,F and S1D,F). FAS is stimulated by SREBP-1c^44^, resulting in favored lipogenesis over lipolysis during infection.

Metformin is a classic AMPK activator and is the most widely prescribed drug for treating hyperglycemia in patients with type 2 diabetes mellitus (T2DM)^45^. It has also been used for prevention in prediabetic populations^46,47^, for avoiding vascular complications associated with T2DM^48^, and for reducing inflammation and hepatic steatosis^49^. Malaria has been associated with T2DM previously^50^. A case-control study conducted in Ghana reported that patients with T2DM had a 46% increased risk of infection with *P. falciparum*^51^ due to impaired defense against liver and blood-stage parasites, decreased T-cell mediated immunity, and increased glucose availability for the parasites^52^. Recent work has shown that maintaining glycemia is essential for tolerance to murine malaria, since severe hypoglycemia contributes to increased brain inflammation and increased lethality of infected mice^53,54^. The use of metformin by T2DM patients significantly reduces the incidence of malaria infection compared to those not on metformin treatment^55^. In addition, metformin has been shown to reduce *P. falciparum* prevalence and to exhibit synergistic effects with antimalarial drugs, including atovaquone^51,56^, and the combination of artesunate/esomeprazole – which was even more effective in reducing glucose levels and attenuating metabolic syndrome symptoms, promoting effective parasite clearance and preventing its recrudescence than the metformin alone^57^.

In this study, we showed that pretreatment with metformin protected *P. chabaudi* infected mice from developing clinically significant malaria. The role of AMPK in infection severity has been previously studied in viral infections^58^ and malaria^59^; however, the latter study was performed during the malaria hepatic cycle. Our study consists of a distinct and novel experimental design, which was used to address the *Plasmodium* intraerythrocytic cycle, where the parasite no longer infects hepatocytes and malarial signs and symptoms are more intense. Thus, we have demonstrated here that malarial disease may be preventable by pretreatment with metformin.

The mechanisms by which metformin pretreatment inhibits *Plasmodium* infection still need to be determined. We suggest, however, that the lipogenic state induced by AMPK downregulation favors *Plasmodium* multiplication and dissemination within the mammal host, while metformin-induced AMPK activation limits the lipid supply to the parasite, thereby impairing infection. Pathogens extensively utilize their hosts’ cellular lipidome pathways, and over millions of years of parasitism they have evolved very sophisticated mechanisms to lengthen their lifespans in their hosts. The acquisition of host lipids is critical in modulating host–pathogen interactions and in improving the pathogens’ virulence^60^. Lipids are an energy source for the pathogens, which cannot synthesize fatty acids, and so they rely on the acquisition of these molecules from the host and subsequent induction of β-oxidation^61^. In the case of *Plasmodium*, acquisition of host lipids is fundamental and particularly important for the intraerythrocytic stage, when this parasite loses the ability to synthesize fatty acids and is only capable of storing lipids within its parasitophorous vacuole^62^. Furthermore, host lipids provide building blocks for pathogen assembly^63–65^ and a convenient carbon source, as has been described for *Mycobacterium tuberculosis*^66,67^, *Salmonella*^68^, *Candida albicans*^69^, and *Cryptococcus neoformans*^70^.

Pretreatment with metformin significantly decreased the concentration of TNFα, IL-1β, and IL-10 in infected compared to noninfected mice (Fig. 8). Metformin has been shown to attenuate proinflammatory response in several diseases^71–73^. Therefore, metformin-induced AMPK activation suppresses the uncontrolled inflammation triggered by the presence of the parasite in the blood, protecting the liver from injury and consequent lipid accumulation, which will culminate with inhibition of the development and multiplication of *Plasmodium* in the blood.

**Figure 9:**
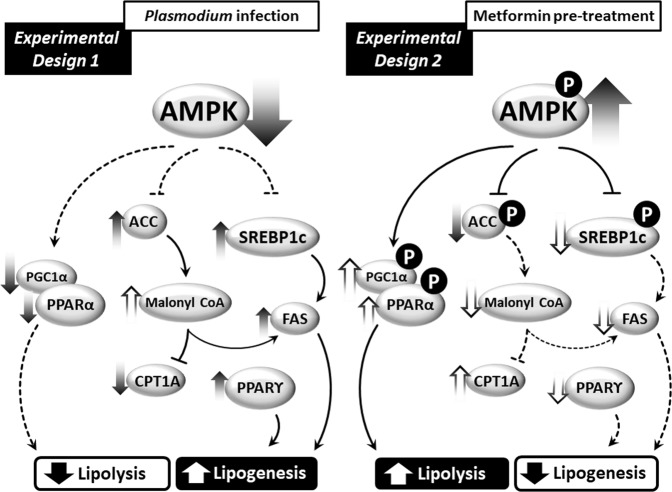
A working model for the role of the enzyme AMPK in regulating lipid metabolism during *P. chabaudi* infection. *P. chabaudi* infection induces a decrease in active hepatic AMPK, which leads to the activation of ACC and SREBP-1c and subsequent stimulation of gene transcription and expression of proteins involved in lipid biosynthesis, such as the enzyme FAS. Concomitantly, there is a reduction of β-oxidation due to the inhibition of the β-oxidation-limiting enzyme CPT1-A and decreased expression of PPAR-α and PGC-1α. Pretreatment with metformin elicits the activation of hepatic AMPK, reversing the lipid metabolism perturbations observed following *Plasmodium* infection and preventing the efficiency of *Plasmodium* infection of mice.

Figure 9 shows a working model of the molecular mechanisms involved in *Plasmodium* infection, indicating a possible route that elicits the accumulation of hepatic lipids during infection in mice. Reduced AMPK activation leads to elevated ACC activation, which stimulates lipid synthesis, and to reduced lipid oxidation due to the inhibition of CPT1-A, a β-oxidation rate-limiting enzyme. Lower AMPK also leads to increased SREBP-1c activation, which stimulates the transcription of genes involved in lipid synthesis, such as FAS. A complementary axis supporting this pathway converges from the increased protein expression of PPARγ, a transcription factor involved on lipid synthesis and decreased phosphorylation levels of PPARα and mRNA expression of PGC-1α, both fatty acid oxidation inducers^74^. Metformin activates hepatic AMPK and inhibits ACC activity, possibly reversing all the signaling pathways that contributed to lipid accumulation in the liver during *Plasmodium* infection in mice. In summary, the data we have presented suggest that AMPK represents an attractive target for new drugs for the prevention and treatment of malaria.

## Materials and Methods

### Parasites and mice

Parasites and mice were obtained from the Laboratory of Ultrastructure and Cellular Biology Hertha Meyer, Carlos Chagas Filho Institute of Biophysics of the Federal University of Rio de Janeiro, in collaboration with Dr. Kildare Miranda. *P. chabaudi* parasites were maintained by successive weekly passages in Swiss mice prior to the experimental infection protocol. Eight-week-old male Swiss mice were kept in a 12 h/12 h light/dark cycle and with water and food *ad libitum*.

### Ethics statement

All animals were treated according to the *Guide for the Care and Use of Laboratory Animals*^75^, and all experimental protocols were approved by the Federal University of Rio de Janeiro and the Center for Health Sciences Animal Care Committee (Protocol IBCCF209-09/16).

### Experimental design 1

Male Swiss mice (~25 g, 8-weeks old) were separated into infected (INF, *n* = 14) and control (CTR, *n* = 14) groups. Infective inocula (1 × 10^6^ RBCs containing *P. chabaudi*) were then prepared and intraperitoneally injected into the INF group. CTR mice received similar inocula but lacking the parasite (1 × 10^6^ uninfected RBCs). After injection, parasitemia was monitored using daily blood smears fixed and stained with the Fast Panoptic method (Laborclin^®^). When parasitemia of INF animals reached 80% (five days after infection), all mice were euthanized in a CO_2_ chamber and blood was collected into vials containing 3% sodium citrate as an anticoagulant agent after cardiac puncture. Livers and retroperitoneal WAT were collected and stored in a liquid nitrogen tank. Blood samples were centrifuged at 500 *g* for 15 min for plasma collection and then stored at −20 °C until needed for analysis.

### Experimental design 2

Male Swiss mice (~25 g, 8-weeks old) were separated into four groups: control (CTR, *n* = 9), infected (INF, *n* = 9), pretreated with metformin (CTR-Met, *n* = 9), and infected and pretreated with metformin (INF-Met, *n* = 9). Groups CTR-Met and INF-Met were pretreated with metformin (150 mg/kg) intraperitoneally once a day for 21 days^76^. Groups CTR and INF underwent similar treatment, but with 0.15 M NaCl instead. Animals were then injected intraperitoneally with the infective inoculum (1 × 10^6^ RBCs containing *P. chabaudi* for groups INF and INF-Met) or uninfected inoculum (1 × 10^6^ healthy RBCs for groups CTR and CTR-Met). Parasitemia was monitored by daily blood smears stained with the Fast Panoptic method (Laborclin^®^). Mice were euthanized when the parasitemia of the INF group reached 80% (five days after infection). Blood and tissue samples were collected and stored using the same procedures as in Experimental Design 1.

### Lipid extraction and analysis

Lipid extraction was performed using a methodology modified from Bligh and Dyer (1959)^77^. Liver and WAT samples (10 mg) were added to a 500 µL of lysis buffer (50 mM Tris-HCl, pH 7.4, 1% NP-40, 250 mM NaCl, 5 mM EDTA, and 50 mM NaF), homogenized, and centrifuged at 2,500 *g* for 5 min. Supernatants were added to glass conical tubes along with a 4 mL of chloroform–methanol–water solution (2: 1: 0.8 mL) and shaken intermittently for 2 h. Extracts were then centrifuged at 730 *g* for 20 min at 4 °C in a High-Speed Refrigerated Centrifuge (Hitachi, Ltd., TO, Japan). The supernatant was collected, and 2 mL of water and chloroform (1: 1 mL) were added, and the solution was centrifuged at 730* g* for 20 min at 4 °C prior to collection of the organic phase (containing lipids). The extracted lipids were analyzed by thin-layer chromatography (TLC) using a hexane/diethyl ether/acetic acid solvent (60: 40: 1 v/v)^78^. To visualize the lipids, plates were immersed in a carbonization solution consisting of 8% CuSO_4_ and 10% H_3_PO_4_ for 10 s and heated at 110 °C for 20 min^79^. Plates were analyzed by densitometry using ImageMaster TotalLab software (TotalLab, Newcastle, UK).

### Colorimetric enzyme assays

Plasma TAG, total cholesterol, and glucose concentrations were determined using commercial enzyme kits (Doles^®^, Goiânia, Brazil) and by following the manufacturers’ instructions.

### Protein concentration determination

Plasma protein concentration was determined by the Lowry method^80^, using bovine serum albumin (0.1%) as a standard.

### RNA extraction and cDNA synthesis

Liver samples (10 mg) were homogenized in 500 μL TRIzol reagent (Invitrogen Corporation, Carlsbad, CA, USA) and centrifuged at 7,500 *g* for 15 min at 4 °C. The supernatant was transferred to microtubes containing 200 μL of chloroform (Merck, Darmstadt, Germany). Samples were shaken for 10 s and centrifuged again at 7,500 *g* for 15 min at 4 °C. Supernatants were collected and transferred to new microtubes containing 200 μL of isopropanol (Merck). Samples were mixed gently by inversion and incubated at room temperature for 10 min and then centrifuged at 7,500 *g* for 15 min at 4 °C. The supernatants were discarded, and the precipitate was resuspended in 200 μL of absolute ethanol. Samples were then centrifuged at 3,000 *g* at 4 °C for 15 min, the supernatants were discarded, and the pellets were resuspended in 30 μL of RNase-free water. The RNA concentration was quantified by spectrophotometry using a NanoDrop (Thermo Scientific, Wilmington, NC, USA).

cDNA synthesis was performed using a High-Capacity cDNA Reverse Transcription kit (Applied Biosystems, Foster City, CA, USA). Reactions contained 1 μg of RNA, 2 μL of reaction buffer, 0.8 μL of 100 µM dNTPs, 2 μL of random primers, 1 μL of 50 U/μL reverse transcriptase, and RNase-free water sufficient for a final volume of 22 μL. The reaction was incubated for 10 min at 25 °C and then at 37 °C for 2 h. Samples were stored at −20 °C until use.

### Quantitative polymerase chain reaction (qPCR)

qPCR was performed using StepOne Real-Time PCR System (Applied Biosystems, Thermo Fisher Scientific, Waltham, MA, USA) and a Power SYBR^®^ Green PCR Master kit (Applied Biosystems, Thermo Fisher Scientific). Primers were custom-designed (Table 1), validated using the Primer-BLAST webpage from the National Center for Biotechnology Information (www.ncbi.nlm.nih.gov/tools/primer-blast), and synthesized by Integrated DNA Technologies (Coralville, IA, USA). 5 μL of the cDNA sample was mixed with 0.9 μL of forward and reverse primers mix (0.05 μg/μL), 0.35 μL of dH_2_O, and 6.25 μL of SYBR^®^ Green Master Mix, reaching a final volume of 12.5 μL. Ninety-six-well optical plates were run in the thermocycler using the following parameters: 50 °C for 2 min, 95 °C for 10 min, and 40 cycles of 95 °C for 15 s, 60 °C for 30 s, and 72 °C for 45 s, followed by a melting curve. Relative mRNA expression was calculated by the 2^−ΔΔCT^ method^81^, and *Rplp0* was used as an endogenous control.

### Immunoblots

Liver samples (50 mg) were homogenized in a lysis buffer (50 mM Tris-HCl, pH 7.4, 1% NP-40, 250 mM NaCl, 5 mM EDTA, and 50 mM NaF) containing protease inhibitor cocktail (catalog number P8340, Sigma Chemicals, St. Louis, MO, USA), and protein was quantified by the Lowry method^80^. Extracts (60 μg) were electrophoresed in 10% SDS-PAGE gels, transferred to nitrocellulose membranes (GE Healthcare Life Sciences, Marlborough, MA, USA), and blocked for 1 h with TBS-Tween (20 mM Tris-HCl, pH 7.5, 500 mM NaCl, and 0.1% Tween 20) containing 3% bovine serum albumin. Membranes were subsequently probed with anti-β-actin monoclonal mouse antibody (1: 1000, Santa Cruz, CA, USA), polyclonal rabbit anti-phospho-AMPK (1: 500, Santa Cruz, CA, USA), polyclonal rabbit anti-AMPK (1: 500, Santa Cruz, CA, USA), monoclonal rabbit anti-phospho-SREBP-1c (Abcam, MA, USA), polyclonal rabbit anti-SREBP-1c (1: 500, Santa Cruz, CA, USA), polyclonal rabbit anti-ACC (1: 500, Santa Cruz, CA, USA), monoclonal rabbit anti-phospho-ACC (1: 500, Abcam), and polyclonal rabbit anti-FAS (1: 500, Santa Cruz, CA, USA), anti-PPARγ (1:1000, Santa Cruz, CA, USA), anti-phospho-PPARα (1:1000, Santa Cruz, CA, USA), and anti-PPARα (1:1000, Santa Cruz, CA, USA) Membranes were incubated for 90 min with anti-rabbit or anti-mouse polyclonal antibody conjugated to horseradish peroxidase (HRP; 1: 20000, Santa Cruz, CA, USA). Immunoreactive proteins were visualized by chemiluminescence using an ECL kit (Amersham Pharmacia, GE Healthcare Life Sciences) and detected with an Image Quant LAS 4000 (GE Healthcare Life Sciences, Marlborough, MA, USA). Densitometric quantitative analysis was conducted using ImageMaster TotalLab software (TotalLab).

### Cytokines measurements

TNFα, IL-1β, and IL-10 cytokines analysis was performed on plasma and liver samples. The protocol used followed the manufacturer’s specifications (R&D, Systems Corporation, Mineapolis, MN). Briefly, 96-well microplates were sensitized with anti-TNFα, IL-1β, and IL-10 monoclonal antibodies, diluted in PBS and incubated for 24 hours at room temperature. The plates were blocked with PBS/4% bovine albumin serum (SAB-Sigma Chemicals, St. Louis, MO, USA) and incubated for 2 hours at room temperature. After following three wash cycles with 0.05% PBS-Tween-20 buffer, samples and standard curve serial dilutions were added to the plates and incubated for 1 hour at room temperature. Thereafter, biotin-conjugated antibodies were added to the plates and incubated for 1 hour. Following, streptavidin peroxidase was added for 20 minutes. After further 3 wash cycles, the 3′, 3′, 5, 5′ tetramethylbenzidine susbtrate (TmB, Zymed) was added to the plates and the reaction was stopped by addition of sulfuric acid 1 M. The plates were read using an ELISA microplate reader at 450 nm.

### Statistical analysis

Statistical analyses were performed using GraphPad Prism software version 6.0 (GraphPad Inc., CA, USA) and included unpaired Student’s *t*-test, one-way ANOVA followed by Dunnett’s multiple comparisons test, and two-way ANOVA followed by Tukey’s multiple comparisons test. Specific statistical analyses are described in the figures’ legends. Results are expressed as mean ± standard error, and differences were considered significant when *p* < 0.05.

## Supplementary information

Supplementary info

## Data Availability

All data generated or analysed during this study are included in this published article (and its Supplementary Information Files).
