# Publisher Correction: Plasmodium Infection Induces Dyslipidemia and a Hepatic Lipogenic State in the Host through the Inhibition of the AMPK-ACC Pathway

**DOI:** 10.1038/s41598-020-62515-9

**Published:** 2020-03-31

**Authors:** George Eduardo Gabriel Kluck, Camila Hübner Costabile Wendt, Guinever Eustaquio do Imperio, Maria Fernanda Carvalho Araujo, Tainá Correa Atella, Isabella da Rocha, Kildare Rocha Miranda, Georgia Correa Atella

**Affiliations:** 1 Laboratory of Lipid and Lipoproteins Biochemistry, Leopoldo de Meis Institute of Medical Biochemistry, Federal University of Rio de Janeiro, Rio de Janeiro, Brazil; 2 Laboratory of Cellular Ultrastructure Hertha Meyer, Carlos Chagas Filho Institute of Biophysics, Federal University of Rio de Janeiro, Rio de Janeiro, Brazil; 3 Laboratory of Translational Endocrinology, Carlos Chagas Filho Institute of Biophysics, Federal University of Rio de Janeiro, Rio de Janeiro, Brazil; 4 Laboratory of Comparative Neurobiology and Development, Carlos Chagas Filho Institute of Biophysics, Federal University of Rio de Janeiro, Rio de Janeiro, Brazil

Correction to: *Scientific Reports* 10.1038/s41598-019-51193-x, published online 11 October 2019

In the original version of this Article, the author Guinever Eustaquio do Imperio was incorrectly indexed. This error has now been corrected.

