# Supplementary material for: Plasmodium Infection Induces Dyslipidemia and a Hepatic Lipogenic State in the Host through the Inhibition of the AMPK-ACC Pathway
Source: Sci Rep. 2019 Oct 11;9:14695. doi: 10.1038/s41598-019-51193-x (PMC6789167; doi:10.1038/s41598-019-51193-x)

Plasmodium Infection Induces Dyslipidemia and a Hepatic Lipogenic State in the Host through the Inhibition of the AMPK-ACC Pathway

George Eduardo Gabriel Kluck[1], Camila Hübner Costabile Wendt[2], Guinever Eustaquio do Imperio[3], Maria Fernanda Carvalho Araujo[1], Tainá Correa Atella[4], Isabella da Rocha[1], Kildare Rocha Miranda[3], Georgia Correa Atella[1*]

Supplementary information

S1A. Protein expression of key enzymes and transcription factors involved in lipid metabolism in the liver of mice infected with *P. chabaudi.* Representative immunoblots with different images exposures from p-AMPK, total AMPK and beta-actin.

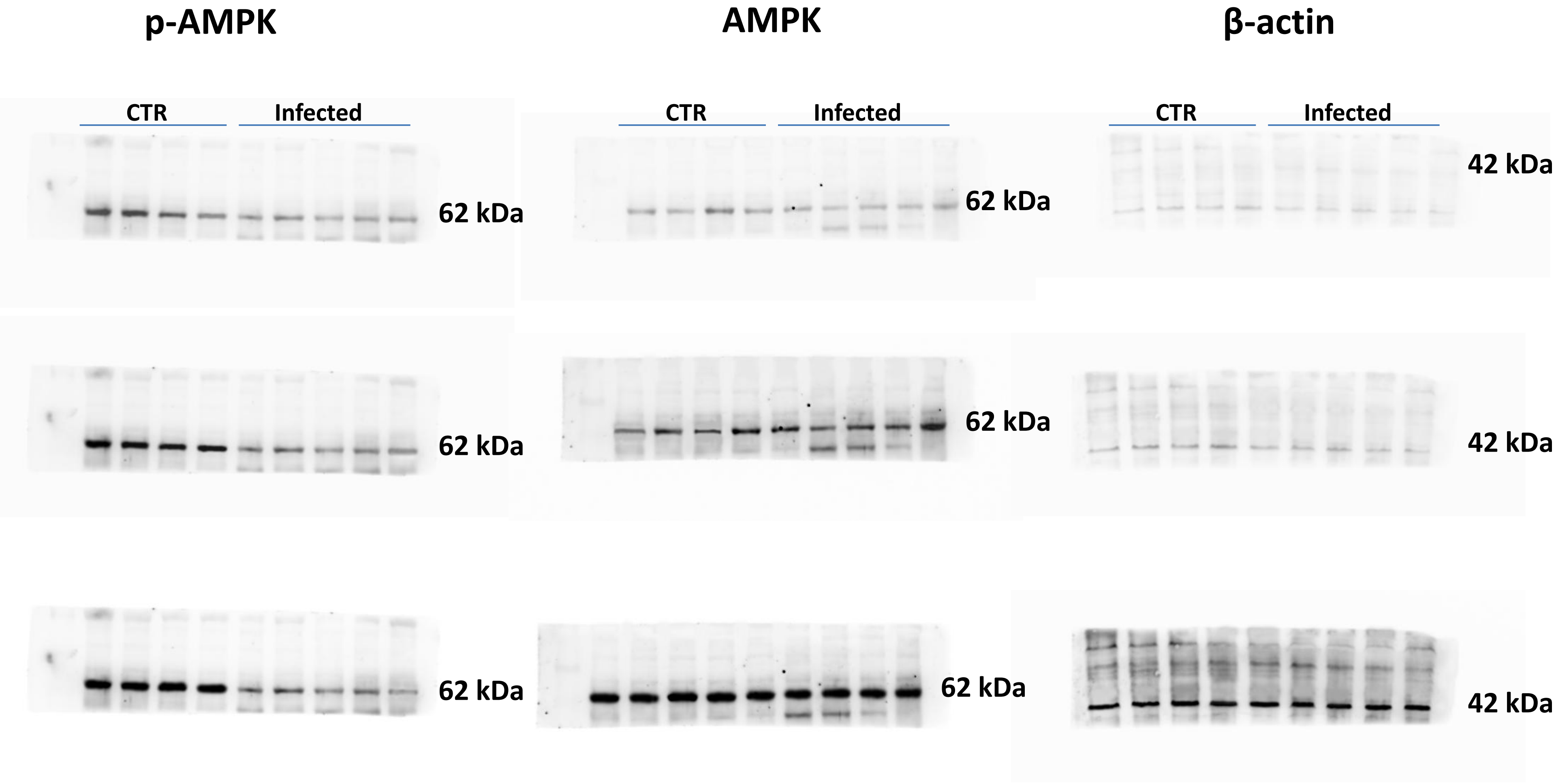

S1B. Protein expression of key enzymes and transcription factors involved in lipid metabolism in the liver of mice infected with *P. chabaudi.* Representative immunoblots with different images exposures from p-ACC, total ACC and beta-actin.

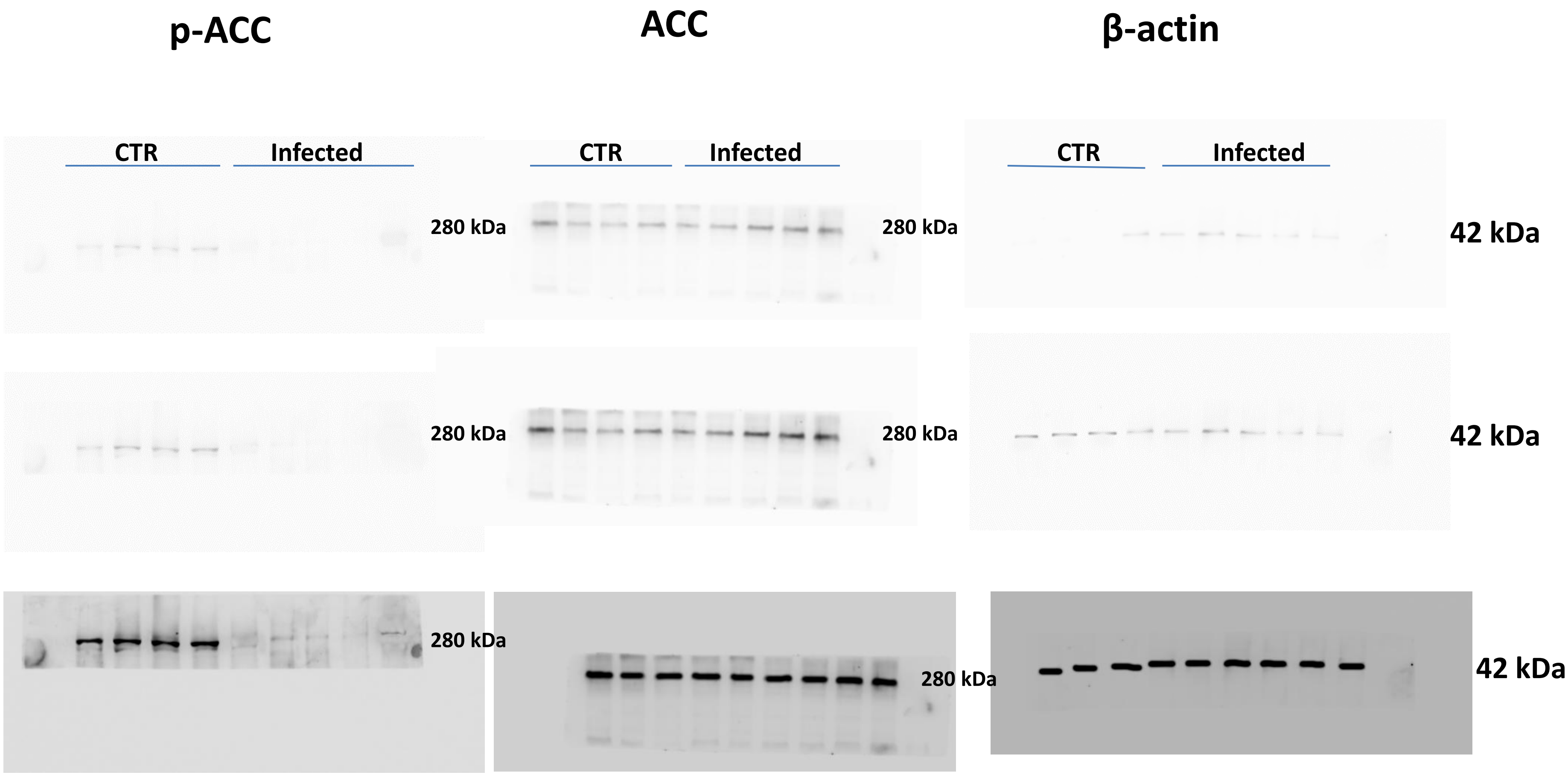

S1C. Protein expression of key enzymes and transcription factors involved in lipid metabolism in the liver of mice infected with *P. chabaudi.* Representative immunoblots with different images exposures from p-SREBP1c, total SREBP1c and beta-actin.

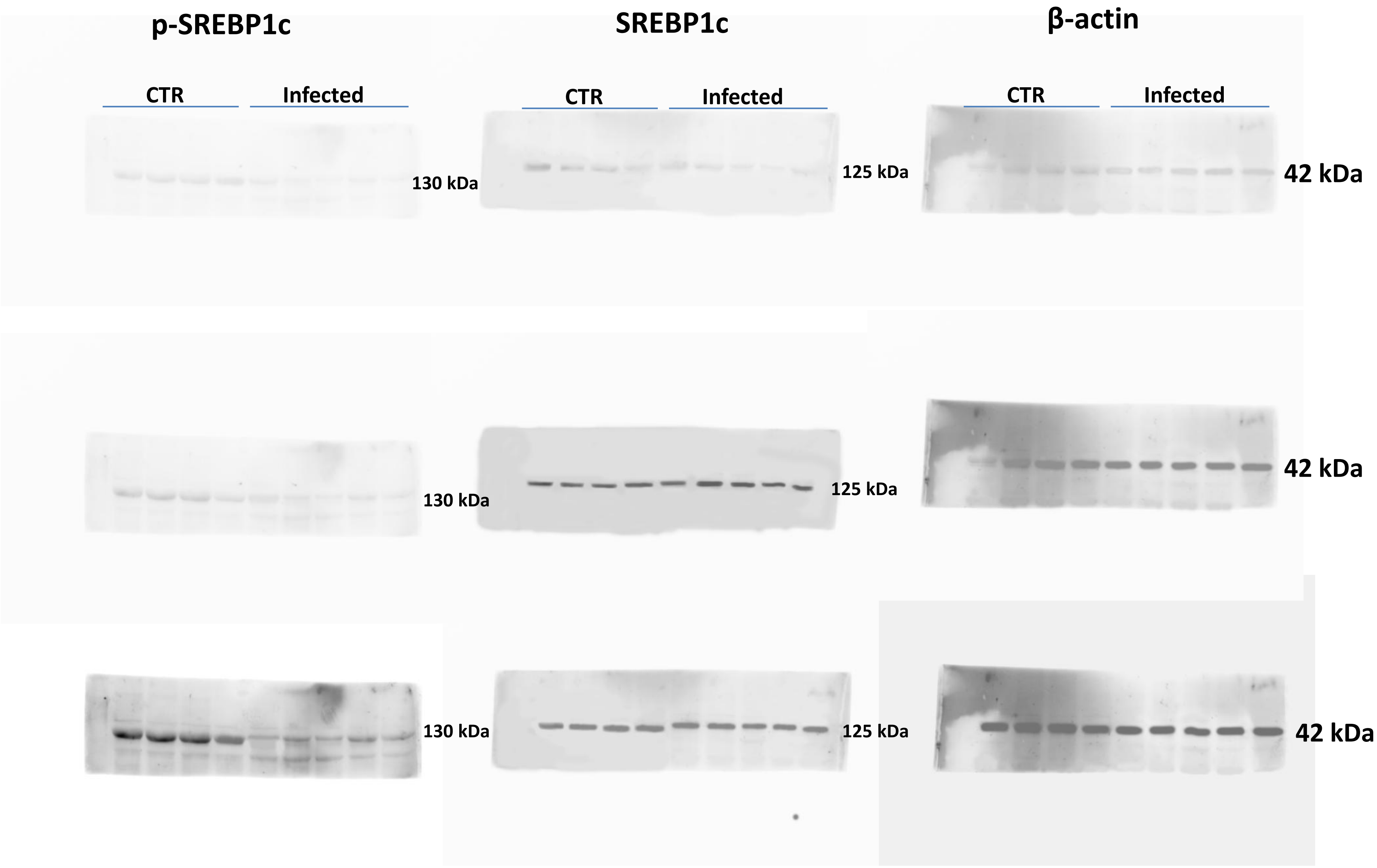

S1D. Protein expression of key enzymes and transcription factors involved in lipid metabolism in the liver of mice infected with *P. chabaudi.* Representative immunoblots with different images exposures from total FAS and beta-actin.

FAS

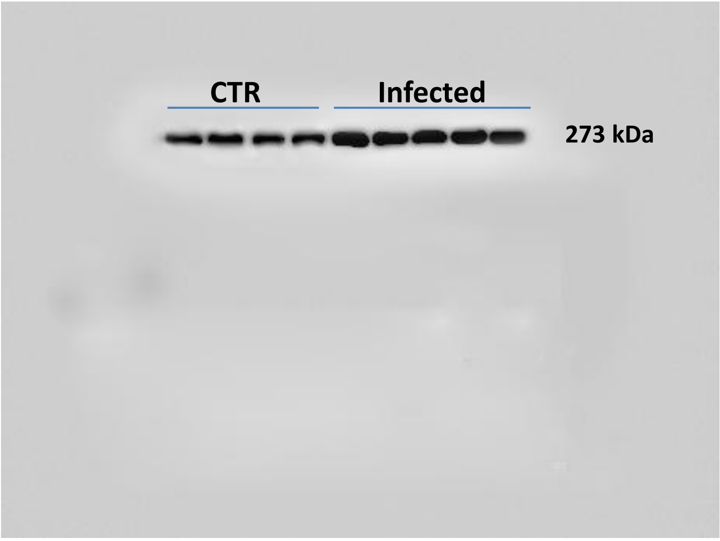

β-actin

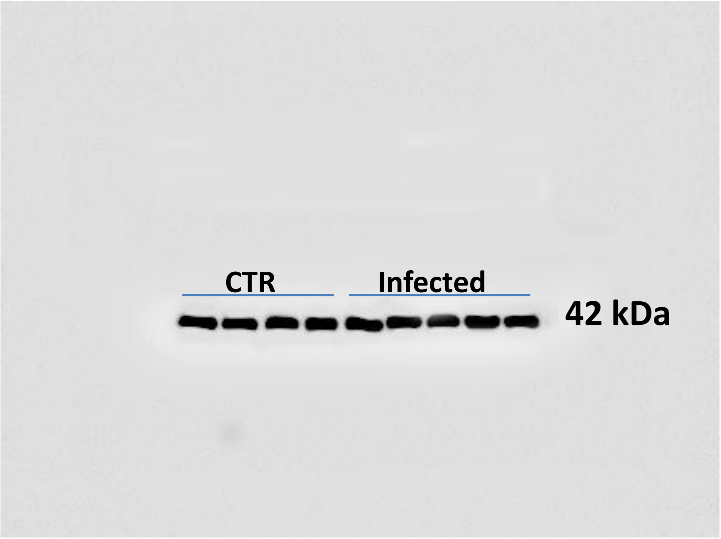

S1E. Protein expression of key enzymes and transcription factors involved in lipid metabolism in the liver of mice infected with *P. chabaudi.* Representative immunoblots with different images exposures from PPARα, PPARγ, and beta-actin.

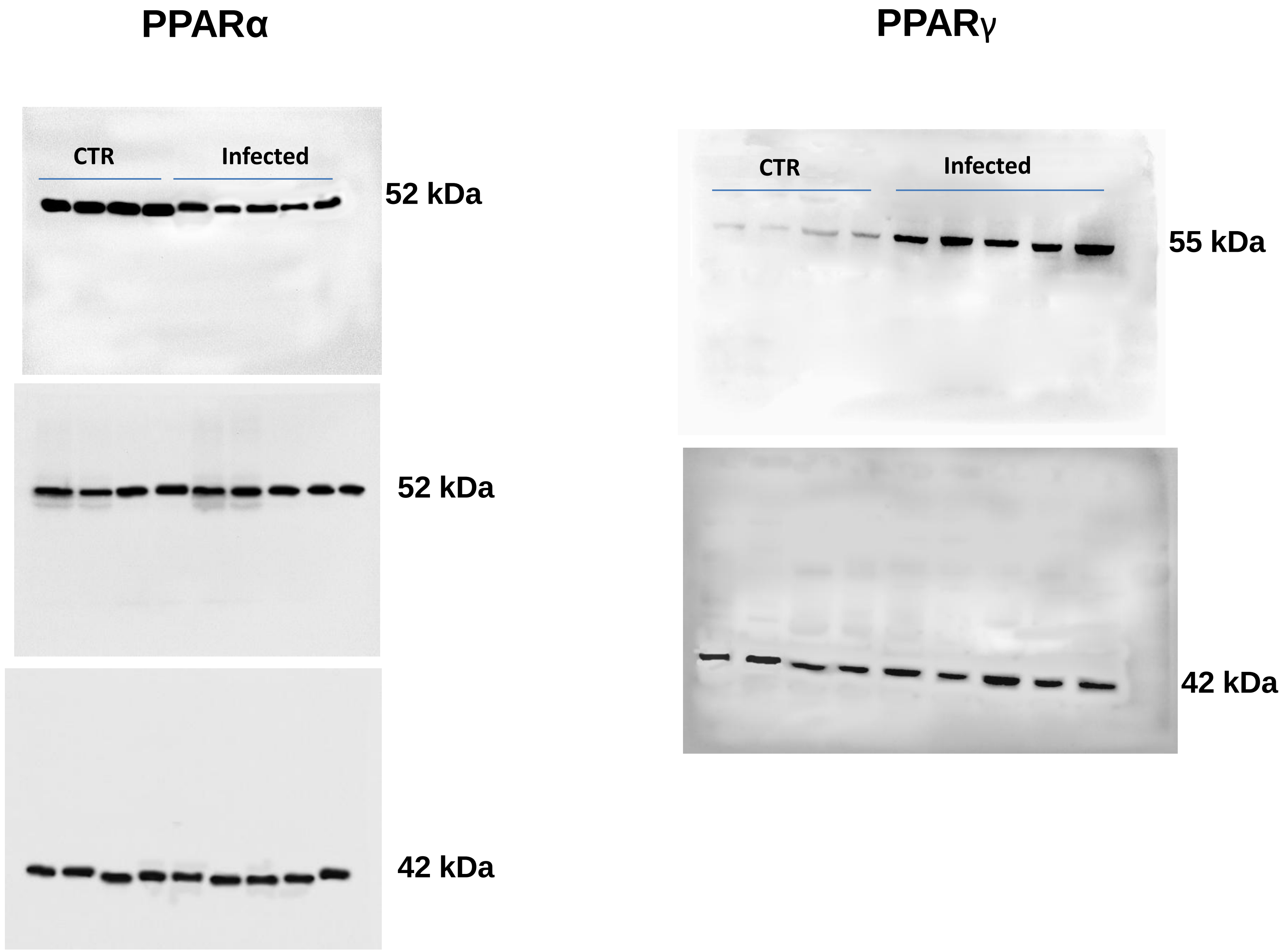

S2A. Protein expression of key enzymes and transcription factors involved in lipid metabolism in the liver of mice infected with *P. chabaudi* and treated with metformin. Representative immunoblots with different images exposures from p-AMPK, total AMPK and beta-actin.

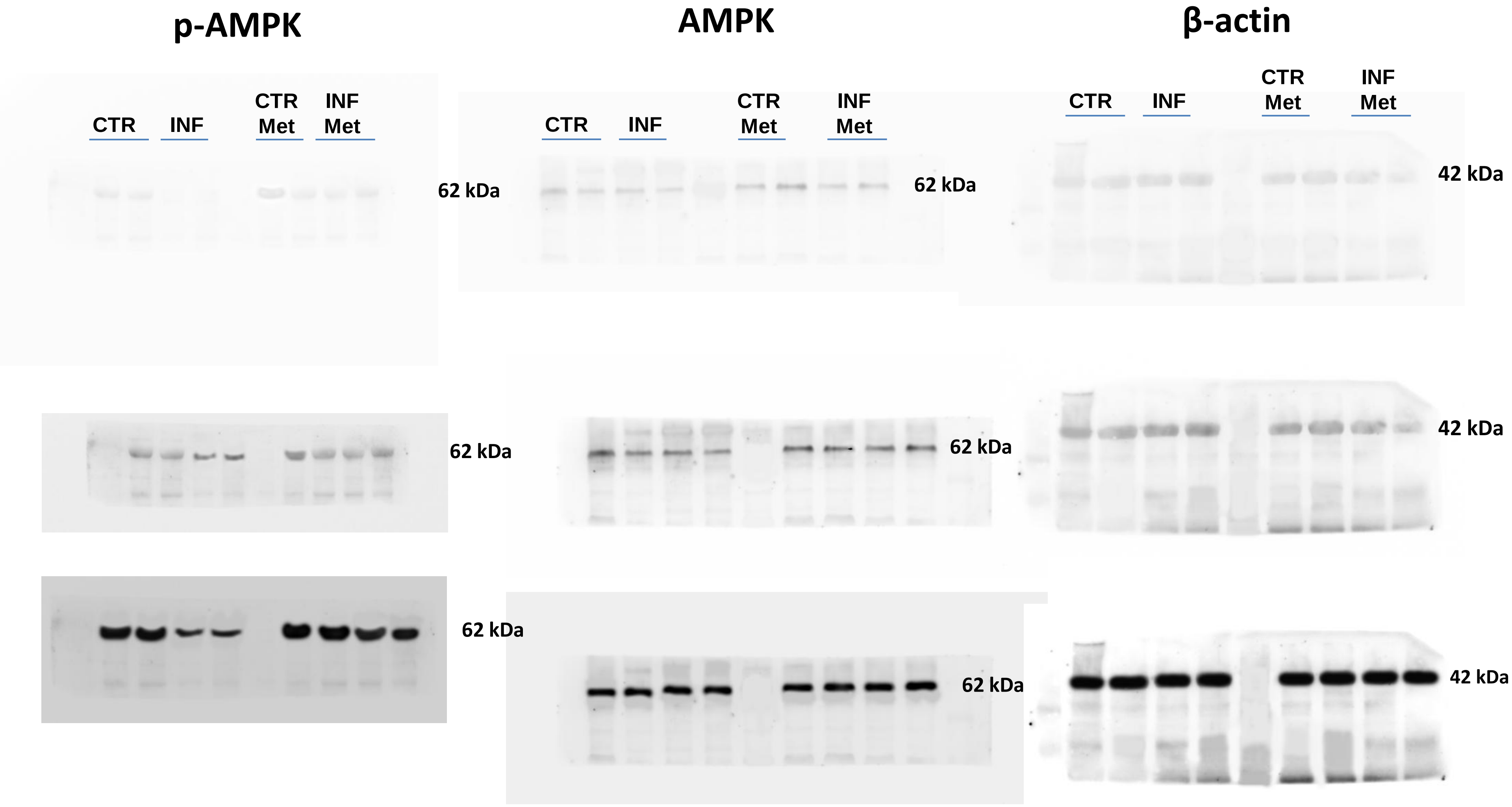

S2B. Protein expression of key enzymes and transcription factors involved in lipid metabolism in the liver of mice infected with *P. chabaudi* and treated with metformin*.* Representative immunoblots with different images exposures from p-ACC, total ACC and beta-actin.

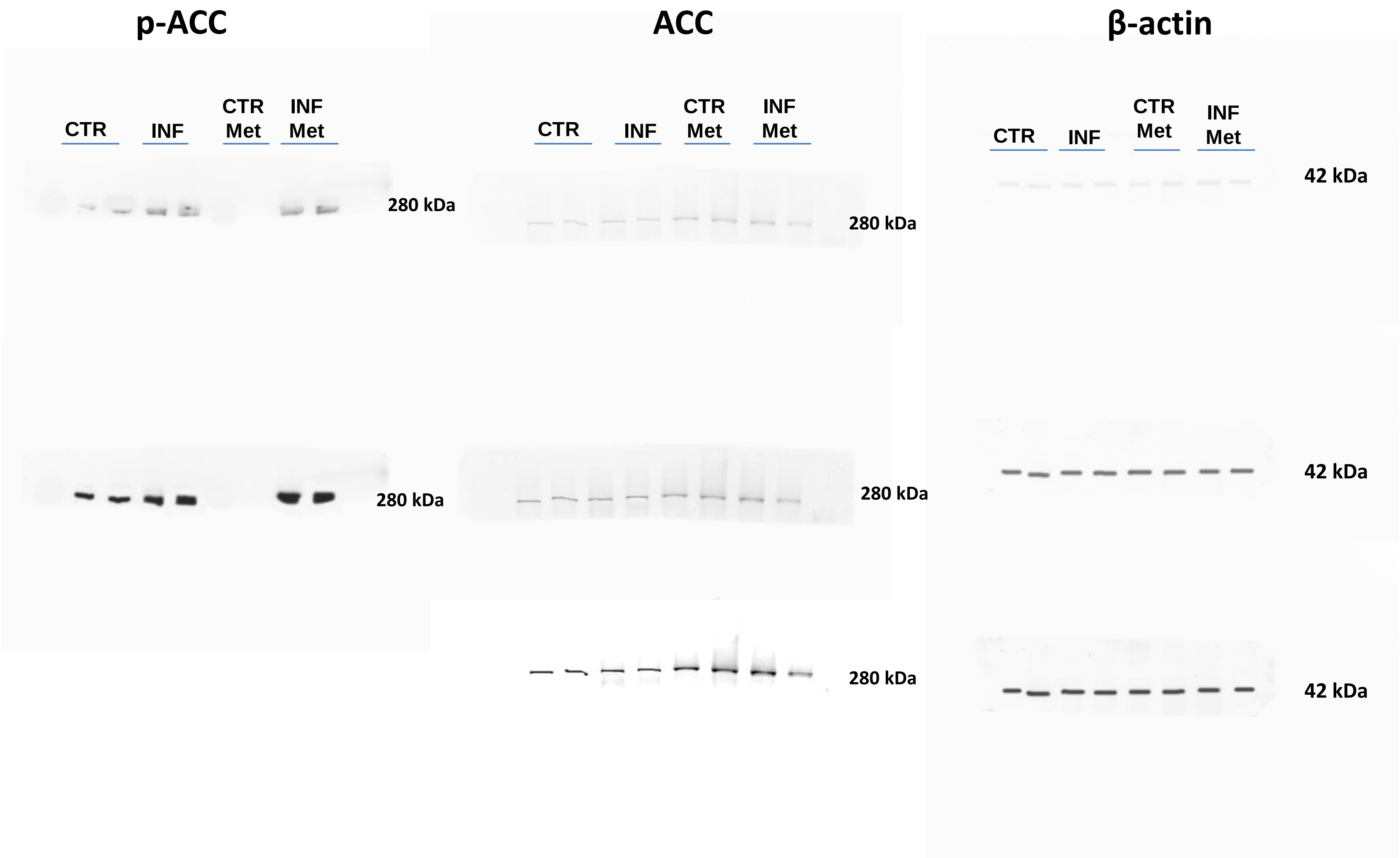

Supplement: Supplementary file 1 — Supplementary info [file 41598_2019_51193_MOESM1_ESM.pdf]
